# The European Rare Diseases Therapeutic Initiative

**DOI:** 10.1371/journal.pmed.0020243

**Published:** 2005-08-23

**Authors:** Alain Fischer, Pascale Borensztein, Claire Roussel

## Abstract

For many rare diseases, doctors have nothing to offer in the way of specific treatments to alter the disease. A new public- private partnership aims to address this problem.

Over 5,000 rare diseases have been described, the vast majority being of genetic origin. Rare diseases affect a small number of individuals—less than one person per 2,000, according to the European definition, or less than 200,000 individuals in the United States, according to the US definition [1].

The few available epidemiological data are too limited to give firm details on the number of patients with a specific rare disease. Some rare diseases, such as cystic fibrosis, are relatively frequent and well known to the general public, but many diseases are extremely rare, affecting only a few patients. Although individually rare, these diseases affect collectively about 30 million Europeans and 25 million North Americans. Such diseases, therefore, represent a major public-health concern, especially considering that a large percentage of these severe diseases are life threatening or cause chronic illness, with a major impact on quality of life. Many of these rare diseases share common characteristics such as a lack of scientific and medical knowledge about the disease, diagnostic tools, and appropriate treatment, leading affected patients to relative isolation.

For many rare diseases with a significant health burden—such as ataxia telangiectasia, Usher syndrome, progeria, and amyotrophic lateral sclerosis—there is simply no specific treatment for the disease. The development of treatments faces two hurdles: the lack of understanding of underlying pathophysiological mechanisms and the lack of interest from the pharmaceutical industry in rare diseases. Indeed, the high cost of drug development, together with the estimated low return on investment (due to very small patient populations), has discouraged the pharmaceutical industry from developing drugs for rare diseases, despite the huge medical need.

## The Need for Incentives

Some hope arose from the “orphan drug” concept (i.e., drugs for rare diseases that have inadequate treatments) and regulations designed to stimulate development of such orphan drugs by providing assistance and economic incentives. The main incentive is probably the promised marketing exclusivity of seven years in the United States [2] and ten years in Europe, beginning on the date of market approval, for products with an orphan designation.

The first country to propose such a legal framework was the US, with the US Orphan Drug Act (1983) [3], followed by Japan in 1993, Australia in 1998, and Europe in 1999 [4]. These orphan drug regulations have proved their success, particularly in certain fields (oncology, immunology, and metabolic disorders) [2,5]. Since the implementation of the US Orphan Drug Act, 1,432 drugs and biological products have been designated as orphan products, and 266 orphan products have received marketing approval from the US Food and Drug Administration. In Europe, only four years after implementation of the European orphan regulation [4], 271 products have been designated and 18 products have received marketing authorization. Moreover, as a result of the orphan regulations, several biotechnology firms have been created to develop treatments for rare diseases.

## Intensifying Therapeutic Research

Although orphan drug regulations have certainly facilitated the development of treatments for rare diseases, major difficulties still persist and additional initiatives are needed. As mentioned, one of the main obstacles is the lack of understanding of the pathophysiology of many rare diseases. Since the identification of therapeutic targets largely depends on the genetic and molecular characterization of the diseases and on the elucidation of biological mechanisms, it is crucial to intensify clinical, genetic, and pathophysiological research on rare diseases [1]. There is, thus, a pressing need to increase the public research effort at national and international levels.

With advances in research, sequencing of the human genome, and development of high-throughput genomic and post-genomic tools, we may expect that the mechanisms underlying many rare genetic disorders will be unraveled in the next few years. For these disorders, therapeutic research also needs to be promoted, including innovative biotechnological research (monoclonal antibodies, cell and gene therapy, and enzyme replacement therapy) as well as classical therapeutic research based on the search for active chemical compounds.

Indeed, even in the field of rare genetic disorders, selection of chemical compounds acting on identified biological targets represents an important objective for drug discovery. Since in most cases pharmaceutical industries will not undertake this primary step, it is important to develop a public-sector interest in doing so.

Two strategies can be exploited to identify active chemical compounds. The first strategy—widely applied by the pharmaceutical industry for drug discovery—is high-throughput screening of several thousands of compounds on biological targets. Such an approach is now possible within the academic sector thanks to the increased availability of public nonproprietary chemical libraries and screening facilities. This strategy requires development of a relevant biological assay that must be adapted to robotization.

The second strategy—a more focused approach—is based on pathophysiological knowledge of the diseases, leading to identification of therapeutic targets. Specifically designing new compounds seems very difficult, given the very high costs involved, but the evaluation of existing chemical compounds with known biological activities represents a very interesting (and much less costly) option for therapeutic research. The thousands of compounds that have been developed by pharmaceutical companies for more common diseases but that were abandoned or failed to achieve registration for several reasons (such as biopharmaceutical properties, toxicity, lack of efficacy, or strategic reasons) represent a treasure worth exploiting. The availability of such compounds could allow us to shortcut the traditional route of pharmaceutical development and evaluate swiftly—at minimal costs—drug candidates for the treatment of rare diseases.

## Toward a Public–Private Partnership

The challenge is thus to manage the interface between pharmaceutical companies and traditional public-sector organizations by establishing a public–private collaboration, leading to the evaluation of these drug candidates in the field of rare diseases. Such a partnership between public research organizations and private companies has now been established. It is called European Rare Diseases Therapeutic Initiative (ERDITI) (http://www.erditi.org), and it has three main objectives: (1) to provide academic teams with facilitated access to available compounds developed by companies, (2) to provide a streamlined facilitated process of collaboration between public and private partners, and (3) to guarantee continuity all the way from preclinical research to development and commercialization of the drug.

ERDITI is sponsored by the European Science Foundation and is coordinated by the French Institute for Rare Diseases Research. This institute is an informal organization, grouping the ministries in charge of research, health, and industry, the French National Institute for Health and Medical Research, the French National Center for Scientific Research, patients' organizations (French Muscular Dystrophy Association and French Rare Diseases Alliance), and public health insurance. Its goal is to foster research on rare diseases in France, but it also works at the European level.

To date, four major pharmaceutical companies involved in research—Aventis, GlaxoSmithKline, Roche, and Servier—and about ten European research institutions or organizations support ERDITI (Box 1). A flexible management and coordination approach has been adopted (Figure 1). Any academic researcher conducting a project on a rare disease, or a group of rare diseases, who is willing to evaluate the therapeutic potential of chosen compounds for preclinical studies simply needs to apply to participate (at http://www.erditi.org). The suitability of the request is assessed by a scientific advisory committee, consisting of European experts from both academic and private sectors. After approval, pharmaceutical company partners are questioned on the availability of molecules belonging to pharmacological classes of interest. If a molecule (or several molecules) is available, a specific agreement is signed between the industry partner and the academic team. Then the industry partner provides reasonable quantities of the molecules required for preclinical studies, together with the necessary information about the molecules. Obviously, transactions with each pharmaceutical company partner are dealt with separately and in confidentiality. It should be emphasized that requests must be based on a pathophysiological hypothesis, and that the partnership excludes high-throughput screening or the building of a common “non-used compounds” library.

**Box 1. European Research Institutions and Organizations Supporting ERDITI**

- The Medical University of Vienna
- Belgium Fund for Scientific Research
- Danish Medical Research Council
- French National Center for Scientific Research
- French National Institute of Health and Medical Research
- German Aerospace Center Project Management Organizations–Health Research
- The Netherlands Organization for Health Research and Development
- Dutch Steering Committee on Orphan Drugs
- The Croatian Academy of Sciences and Arts
- The Spanish Institute for Research on Rare Diseases
- Slovak Academy of Sciences

**Figure 1:**
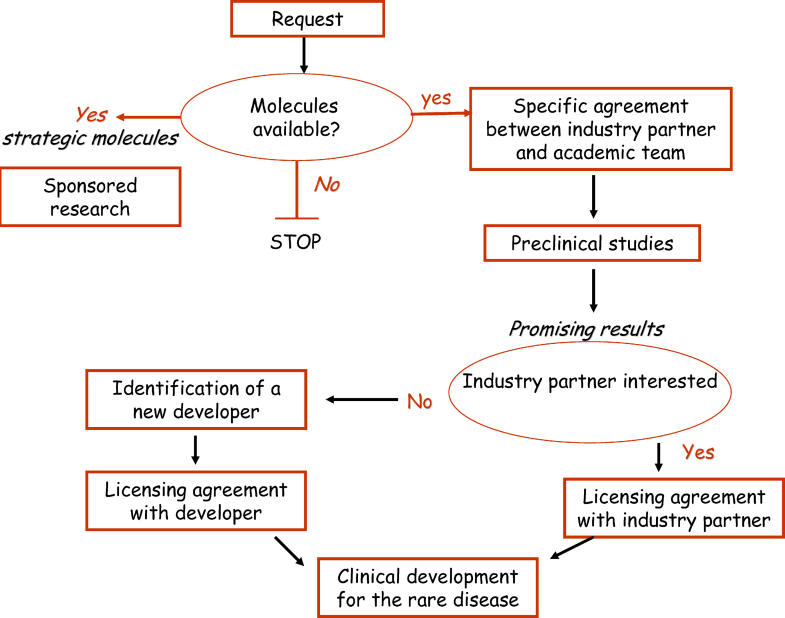
How ERDITI Functions as a Public–Private Partnership The working procedure guarantees continuity all the way from research to development of a drug. After the ERDITI scientific advisory board's assessment of the request, industry partners are questioned about the availability of molecules belonging to the pharmacological classes of interest. Several molecules may be of strategic interest for the industry partner, and sponsored research should be negotiated directly outside the framework of this working procedure. If there are no molecules available, the process ends. If molecules are available, a specific agreement is signed and the industry partner provides molecules required for preclinical studies. If promising results arise from preclinical studies, the lead academic partner asks the industry partner whether it wishes to exercise the option to further develop the drug. The industry partner may decide to exploit the results and develop one of the molecules through a worldwide exclusive license. Alternatively, the industry partner may decide not to exploit the results and grant necessary license rights to a third party for the development of the molecule for treating the rare disease.

## A Charter of Collaboration

The ERDITI partnership is based on a charter of collaboration, including a standard agreement that describes the objectives of the research and defines the framework for the transfer and use of compounds. It also defines the rights and obligations of each party in terms of protection of information, intellectual property, and industrial property.

Obviously, intellectual property rights had to be clarified between academic and industrial partners. It has been agreed that the results originating from the study in the field of rare diseases will be the sole property of the academic partner but may be freely used by the industry partner for internal research purposes. On the other hand, when research on the molecules supplied by the industry yields results that are applicable outside of the field of rare diseases, these results shall be the pharmaceutical company's sole property. In this case, if the industry partner wants to commercially exploit these results outside the field of rare diseases, a royalty is negotiated by the public-sector partner.

Some orphan products have proved their profitability

Another key point of the agreement is to guarantee that the project will continue from research to development in cases where preclinical studies uncover the potential of a chemical compound for treating a rare disease. Indeed, the industry partner who has rights to this drug will either develop the drug for the rare disease indication through a worldwide exclusive license agreement, or it will allow its development by an academic team or a third party (in which case the industry partner grants the necessary license rights for the drug's development by a third party).

## How Does Industry Benefit?

One may wonder, what could be the benefits for industry of entering into such a partnership? For a few years now, some pharmaceutical companies have shown an interest in rare diseases for four reasons. Firstly, they seek an image of a “socially responsible company.” Secondly, some orphan products such as imatinib mesylate (Gleevec) or enzyme replacement therapies have clearly proved their profitability. Thirdly, research on rare diseases may be profitable for more common diseases—several rare diseases might represent valuable proof-of-concept models, and their study may repay research efforts by leading to the discovery of drugs for the treatment of more frequent diseases. Finally, ERDITI offers drug companies the chance to give new indications to abandoned compounds.

**Related Web Sites**

- European Medicines Agency Committee for Orphan Medicinal Products: http://www.emea.eu.int/htms/human/comp/compsumop.htm
- US Food and Drug Administration Office of Orphan Products Development: http://www.fda.gov/orphan
- Orphanet (a database dedicated to information on rare diseases and orphan drugs): http://www.orpha.net/

We must make it clear that the industrial partners are not forced to supply compounds or to give any reason for refusal. The authenticity of their commitment will be measured in the future only by concrete results, which means by the extent to which they make compounds available for therapeutic research projects. However, the commitment of the four pharmaceutical companies already involved with ERDITI—who are willing to take up the challenge and to open their compound libraries to academic therapeutic research—really raises hope for future development of new drugs.

## The Coming Challenges

To ensure the success of this public–private partnership, it needs to be better publicized within the scientific community. Indeed most European scientists are not aware of this initiative, because of a lack of information about it within universities and research institutions.

The issue of funding for research projects is also an important challenge. ERDITI does not provide any direct additional funding mechanism for research, and so researchers must obtain funds from their customary sources for their preclinical projects. In France, some specific funding incentives have been established but this effort must be strengthened. At the European level, the challenge will be to establish and fund a public–private platform for rare diseases that achieves two things: (1) the ability to identify and fund promising preclinical projects and (2) the development of clinical multicentered projects through provision of the necessary expertise and funding.

Once we have shown that ERDITI is a feasible initiative, we intend to open ERDITI to other pharmaceutical companies or biotechnology societies, and to broaden its scope to a more global partnership.

## Abbreviation

ERDITI: European Rare Diseases Therapeutic Initiative

## References

[pmed-0020243-b1] Van Weely S, Leufkens HGM, Kaplan W, Laing R (2004). Priority medicines for Europe and the world—A public health approach to innovation. Orphan diseases.

[pmed-0020243-b2] Haffner ME, Whitley J, Moses M (2002). Two decades of orphan product development. Nat Rev Drug Discov.

[pmed-0020243-b3] US Food and Drug Administration (1983). The orphan drug act. www.fda.gov/orphan/oda.htm.

[pmed-0020243-b4] European Parliament Council of the European Union (2000). Regulation (EC) No 141/2000 of the European Parliament and of the Council of 16 December 1999 on orphan medicinal products. Official J Eur Communities.

[pmed-0020243-b5] European Agency for the Evaluation of Medicinal Products (2003). Report on the first 3-year mandate of the Committee for Orphan Medicinal Products (COMP): 2000 April–2003 April. http://www.emea.eu.int/pdfs/human/comp/911803en.pdf.

